# How cyanobacteria pose new problems to old methods: challenges in microarray time series analysis

**DOI:** 10.1186/1471-2105-14-133

**Published:** 2013-04-21

**Authors:** Robert Lehmann, Rainer Machné, Jens Georg, Manuela Benary, Ilka Axmann, Ralf Steuer

**Affiliations:** 1 Institute for Theoretical Biology, Humboldt University Berlin, Invalidenstraße 43, D-10115, Berlin, Germany; 2 Institute for Theoretical Biology, Charité Universitätsmedizin, Invalidenstraße 43, D-10115, Berlin, Germany; 3 Institute for Theoretical Chemistry, University of Vienna, Währingerstraße 17, A-1090, Vienna, Austria; 4 Faculty of Biology and Freiburg Initiative in Systems Biology, University of Freiburg, Schänzlestraße 1, D-79104, Freiburg, Germany; 5 Global Change Research Center – CzechGlobe, Academy of Sciences of the Czech Republic, Zamek 136, CZ-37333 Nove Hrady, Czech Republic

## Abstract

**Background:**

The transcriptomes of several cyanobacterial strains have been shown to exhibit diurnal oscillation patterns, reflecting the diurnal phototrophic lifestyle of the organisms. The analysis of such genome-wide transcriptional oscillations is often facilitated by the use of clustering algorithms in conjunction with a number of pre-processing steps. Biological interpretation is usually focussed on the time and phase of expression of the resulting groups of genes. However, the use of microarray technology in such studies requires the normalization of pre-processing data, with unclear impact on the qualitative and quantitative features of the derived information on the number of oscillating transcripts and their respective phases.

**Results:**

A microarray based evaluation of diurnal expression in the cyanobacterium *Synechocystis sp.* PCC 6803 is presented. As expected, the temporal expression patterns reveal strong oscillations in transcript abundance. We compare the Fourier transformation-based expression phase before and after the application of quantile normalization, median polishing, cyclical LOESS, and least oscillating set (LOS) normalization. Whereas LOS normalization mostly preserves the phases of the raw data, the remaining methods introduce systematic biases. In particular, quantile-normalization is found to introduce a phase-shift of 180°, effectively changing night-expressed genes into day-expressed ones. Comparison of a large number of clustering results of differently normalized data shows that the normalization method determines the result. Subsequent steps, such as the choice of data transformation, similarity measure, and clustering algorithm, only play minor roles. We find that the standardization and the DTF transformation are favorable for the clustering of time series in contrast to the 12 m transformation. We use the cluster-wise functional enrichment of a clustering derived by LOS normalization, clustering using flowClust, and DFT transformation to derive the diurnal biological program of *Synechocystis sp.*.

**Conclusion:**

Application of quantile normalization, median polishing, and also cyclic LOESS normalization of the presented cyanobacterial dataset lead to increased numbers of oscillating genes and the systematic shift of the expression phase. The LOS normalization minimizes the observed detrimental effects. As previous analyses employed a variety of different normalization methods, a direct comparison of results must be treated with caution.

## Background

Photosynthetic organisms such as cyanobacteria have been shown to employ complex diurnal regulatory patterns to prepare the organism for the light period [1-3]. The extent, purpose and mechanism of diurnal and circadian oscillations have been reported to differ significantly among various cyanobacterial species. In particular, the reported estimates of the number of oscillating transcripts differ strongly between studies, ranging between 9-80% of protein-coding genes in microarray time series [4-8]. Random insertion of a luciferase reporter system indicated that up to 100% of genes may be under circadian control [1,9]. Although microarray technology is a powerful genomic approach to quantify the expression levels of large numbers of genes simultaneously, there are technical limitations which significantly complicate the quantification and interpretation of such global transcriptional rearrangements. Here, we consider a large number of combinations of methods required in a typical microarray analysis pipeline to evaluate the impact of each step on the results. In addition to time series-specific descriptors, clustering is used due to its importance as tool for biological interpretation of microarray data.

Microarray platform-inherent technical limitations cause the resulting data to contain systematic or random technical variation in addition to the biological variation of interest [10]. Differences in the distribution of the measured fluorescence values are commonly attributed to variations in the quality of RNA extraction (experimental variation) and of individual arrays (technical variation). Based on assumptions about biologically plausible variation, a range of normalization methods attempt to reduce the technical variation between chips. The expected amount of change in gene expression is a crucial element in the design of normalization methods. This can be a hen-egg-problem in less well studied experimental systems, for which little or no information is available on the expected global remodeling of the transcriptional landscape. Various normalization methods have been employed in previous studies to describe the transcriptional landscape in cyanobacteria. A combination of LOESS and quantile normalization was used by Vijayan *et. al*[11,12]. While spike-in standards were incorporated in these studies, normalization was performed without application of this additional information. Kucho *et al.*[4] and Straub *et al.*[8] employed LOWESS normalization. A modified LOWESS normalization was used in the work of Stöckel *et al.*[3]. As described in more detail by Calza *et al.*, the assumption of constant expression for traditional housekeeping genes does not hold under all conditions. Considering the high percentage of diurnally varying genes in cyanobacteria [9] including central cellular processes such as translation [3], an a priori definition of housekeeping genes is not possible for cyanobacteria. Correspondingly, reports employing housekeeping gene- based normalization are not known to the authors.

It is known that the application of such global normalization methods has significant impact on subsequent analyses, in particular when some of the underlying assumptions on data structure are not or only partially fulfilled [13]. Global normalization methods may change the set of differentially expressed genes [14,15] or lead to significant changes in the observed correlation between genes [16,17]. While cross-validation of expression measurements can be used to discover methodological problems [18], the lack of diurnal expression datasets from alternative techniques, such as RNA-Seq, impedes such verification in the case of cyanobacteria. These observations raise the question, how normalization and other preprocessing steps affect commonly used descriptors for periodic expression, e.g., the number of oscillating genes (by tests of significance of oscillation) and the circadian phase of peak transcript levels [11,19,20]. Such phase information is usually used to derive a temporal order of the observed processes. It is, therefore, of paramount importance to prevent systematical errors in the primary phase information.

In addition to the normalization steps, microarray analyses require a transformation which accounts for its semi-quantitative nature. Calibration methods for sequence-dependent hybridization energies and unspecific cross-hybridizations have been proposed [10,21,22], but are not yet an established standard and so far only implemented for Affymetrix arrays. The interpretation of microarray data in terms of absolute mRNA copy numbers is currently not possible. Instead, data transformations are used to normalize a given transcript time series to the mean value or to the distribution of fluorescence intensities: the fold-change or *log*_2_ mean ratio transformation (in the following: 12 m) removes the mean, while standardization (z-score transformation, in the following: std) additionally normalizes the standard deviation in order to focus on the pattern of change rather than its amplitude [23]. We also consider the discrete Fourier transformation (DFT) in the context of data transformation. The removal of the first DFT component results in a normalization by the expression mean in the 12 m, and an amplitude scaling serves to de-emphasize the amplitude [24], comparable to std. Notably, only this transformation from time to frequency space considers explicitly the time series character of the data.

The biological interpretationof microarray data is possible only after the application of the transformation and normalization. Due to its high-dimensional nature, a standard step in the interpretation of microarray data is clustering. A variety of clustering algorithms have been proposed, making it necessary to systematically evaluate the performance on gene expression data [25,26]. However, due to the diversity of the data domain, a recent work concluded that the choice of a clustering algorithm might depend on the specific experiment [26]. In the case of time series analysis, it has been noted that most clustering algorithms do not consider the pattern of change over time, but treat each sample independently of the temporal order. An increasing number of algorithms propose solutions to this issue [27-31], but there is no accepted standard. An interesting approach specifically designed to cluster periodic time series has recently been proposed for the analysis of respiratory oscillations in budding yeast culture [24]. Here, the DFT of the time-series was clustered with a model-based algorithm that uses t-distributions as a model (flowClust [32]). However, the impact of data transformation and normalization of time resolved microarray data, the clustering algorithm, and the similarity measure on the corresponding clustering result have not been fully described.

Several studies on various model organisms have reported that accepted standard normalization methods lead to inaccurate results under certain experimental conditions. A recent study of a human B cell line verified an increase of the global mRNA abundance per cell. This violation of a common assumption for normalization challenges the conclusions of a wide range of studies (Lovén2012). Furthermore, a work from Machné and Murray [24] compares two independent measurements of the oscillatory transcriptional changes of budding yeast under continuous culture conditions. Due to the global nature of the observed oscillations, an alternative normalization scheme was employed to avoid detrimental effects of standard methods. This normalization method is included in this study. We therefore expect, that the conclusions drawn from our study are also valid for other model systems. However, cyanobacteria are a highly specific model system featuring, e.g., a small number of genes with a high fraction of diurnally oscillating genes. Therefore, we address the case of cyanobacteria with this systematic analysis of normalization methods and demonstrate how to circumvent problems while analyzing diurnal expression data.Ť

The quantification of diurnal expression in the cyanobacterium *Synechocystis sp.* PCC 6803 using microarray technology poses new problems for old methods of data normalization, transformation and clustering. We compare four multi-array normalization methods and three data transformations with respect to diurnal expression oscillation strength and phase. Furthermore, we use a variety of clustering algorithms to examine the global expression landscape. The results of seven clustering algorithms are integrated to verify whether and how normalization shapes the results of downstream analyses. Our analysis demonstrates that normalization methods have significant impact on the estimated number and phases of oscillating transcripts, with major consequences for subsequent analysis and biological interpretation. We identify LOS normalization as the preferable method.

## Results and discussion

### A diurnal trend in the total chip signal

**Figure 1:**
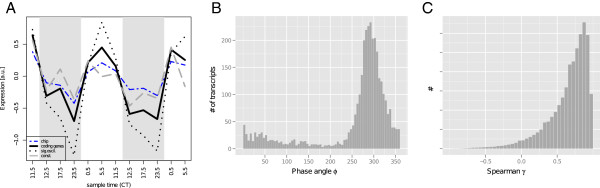
**Oscillation of the unprocessed total signal. A**) Prior to any pre-processing, the mean transcript abundance for all genes on the chip (blue dashed) and all 3347 protein-coding genes (black solid) exhibits diurnal oscillations. Significantly oscillating genes (*p*_*osc*_ < 0.05) resemble the oscillation of the total intensity (black dotted), whereas the non-significantly oscillating genes (*p*_*osc*_ > 0.05) exhibit show increased expression over the day and a peak at 17.5 CT (gray dashed). **B**) The majority of genes exhibit a phase angle *ϕ* in the range of 250−350 corresponding to expression over the day. **C**) The histogram of Spearman correlation coefficients *γ* between all pairwise combinations of the 3447 protein coding genes shows that most genes strongly correlate. Only a small amount of pairs is uncorrelated or anti-correlated.

Cultures of the cyanobacterium *Synechocystis sp.* strain PCC 6803 were synchronized with three cycles of light/dark (LD) 12 h:12 h. During the fourth cycle, six samples were taken in two biological replicates, yielding 12 microarrays. Since the biological replicates were obtained from independent culture flasks, their information content complements each other. In order to utilize the entirety of the available information, we concatenated the two replicates into one time series encompassing 12 points over two consecutive days and comprising 3,347 protein-coding genes, which served as the starting point for further analyses. Sampling times are given in hours of circadian time (CT), which defines light onset as time point 0 h. A diurnal pattern of the total microarray signal is observed in the raw data (Figure 1A), despite the application of similar amounts of RNA (1.5 *μ*g) to each individual chip as in most microarray protocols. Such a diurnal variation of the total mRNA amount has not been reported along with genome-wide expression time series of other cyanobacterial species. However, we recently observed a similar trend in transcriptome time-series of budding yeast respiratory oscillations [24]. We provide plausible interpretations of this observation in the conclusion.

To characterize the periodicities present in the unnormalized data set, we calculated the phase of peak transcript levels and amplitudes for all protein-coding transcripts from the DFT component corresponding to the two LD cycles. Since our samples were taken at non-equidistant sampling intervals, the phases do not linearly correspond to the time domain, but reflect accurately the temporal sequence of transcript level peaks. The significance of periodic transcript levels (*p*_*osc*_) was calculated from a permutation-based background model [19,20,24]. The majority of transcripts peak at phases 250-350° (Figure 1B), corresponding to an expression during the light phase. Strong oscillators (*p*_*osc*_<0.05) reflect the observed global trend, while weak oscillators contain both this global trend and additional peaks at CT17.5, i.e., during the dark phases (Figure 1A). These observation indicate that central assumptions of several common normalization methods may be violated. On the other hand, the additional peaks at CT17.5 may reflect technical rather than true biological variability or represent a mixture of night-activation of gene expression with a global trend, where all transcripts are present at higher levels. Prior to further analysis, the dataset needs to be normalized to distinguish array-to-array noise from true biological signal.

### Normalization leads to changed diurnal expression times

We tested the impact of four normalization methods which have either been previously used to analyze the temporal expression organization in cyanobacterial species [11,12,33] (median polishing, quantile normalization) or have become an established standard [34] (cLOESS). Additionally, we tested a recently proposed procedure which employs a set of least variant genes as reference set for LOESS smoothing [13]. Importantly, the least variant genes method was modified by using the significance of periodicity (*p*_*osc*_, as above) in the raw data to define a least-oscillating set (LOS) of reference genes [24]. Comparison of the periodicity descriptors from unnormalized and normalized data showed that the number of significantly diurnal transcripts was strongly affected, e.g., a cut-off *p*_*osc*_<0.05 retrieved 25% of all transcripts from raw data, 58% from median polished, 60% from quantiles-normalized, 64% from LOS-normalized and 35% from cLOESS-normalized data. At a very conservative cut-off of *p*_*osc*_<0.001, the number of significant oscillators in cLOESS (1.7%) decreased below the level of raw data (raw: 2.2%; quantiles: 4.4%; median polishing: 4.9%; LOS: 7.8%).

**Figure 2:**
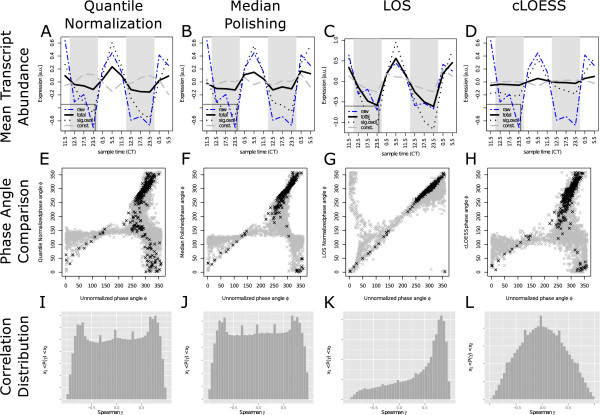
**Normalization changes phase angles and expression correlation.** Systematic comparison of important properties of the expression profile set after normalization with different methods. Columns one to four correspond to the methods quantile normalization, median polishing, LOS, and cLOESS, respectively. Rows one to three correspond to plots of prominent average expression profiles, expression phase comparisons, and pairwise correlation distributions. The mean expression profiles for different gene groups illustrate the impact of normalization methods. A comparison of the unnormalized mean expression profile of all genes (dashed blue) with the normalized mean over all genes (black solid), significantly oscillating genes (*p*_*osc*_ < 0.05 in unnormalized data - black dotted) and not oscillating genes (*p*_*osc*_ > 0.05 in unnormalized data - gray dashed) is shown in panel **A** to **D**. The time of maximal expression in oscillatory profiles, measured using the Fourier transformation, is frequently altered by the normalization method. Panel **E** to **H** show the comparison between expression phases observed in the unnormalized (x-axis) versus normalized (y-axis) data. Profiles with significantly oscillating expression (*p*_*osc*_ < 0.05) are shown in black, whereas weak or non-oscillators are shown in gray (*p*_*osc*_ > 0.05). The histogram of pairwise Spearman correlation coefficients between expression profiles as proxy of the diversity of the global expression landscape is shown in panels **I** to **L**.

While such numbers are interesting to illustrate the extent of transcriptional remodeling, the goal of a microarray analysis is to obtain a temporally resolved picture of the transcriptional landscape. Commonly, the time-series is reduced to a phase angle corresponding to the time during the course of a day where a transcript’s level peak. Thus, we tested the agreement of phase angles *ϕ* between unnormalized data and each normalization (Figure 2E– 2H). A systematic deviation of strong oscillators (*p*_*osc*_<0.05) from the diagonal can be observed for all but the LOS-normalized data. The deviation follows a strong systematic trend of the weakly or non-oscillatory transcripts (*p*_*osc*_>0.05) towards earlier phases of transcript peaks. LOS-normalization has an opposite effect only on the weak oscillators, and shifts them systematically towards later phase, while strong oscillators remain unaffected. Under the assumption that technical noise is independently identically distributed amongst the individual samples (microarrays) of a time series, the removal of such noise contributions should not alter the observed phase of a periodic signal or introduce oscillatory behavior. Since quantile normalization, median polishing and cLOESS compensate for the observed global oscillatory trend, an anti-phase oscillation is introduced into weak oscillatory profiles leading to the large number of genes with phases <*ϕ* 125°, which corresponds to expression during the night. In contrast, phases of weak oscillators are shifted towards the day time by LOS normalization. These systematic shifts percolate into the mean profiles of our set of strong and weak oscillators. While quantile normalization, median polishing and cLOESS all enhance the night-peak and remove the global trend from weak oscillators, the LOS normalization has the opposite effect, i.e., it reinforces the global day-peak and removes the night peak from the mean time courses of all weak oscillators (Figure 2A– 2D). Additionally, cLOESS normalization severely dampens the periodicity of all genes, explaining the decrease in the number of significant oscillators at conservative cut-off thresholds.

To better understand the effects of the different normalization methods, we chose another way of characterizing the data, i.e., the pairwise correlation between expression profiles. Before normalization, the distribution of the pairwise Spearman correlation (Figure 1C) is unimodal with a pronounced peak at 0.8 attesting a very high degree of correlation without significant uncorrelated or anti-correlated pairs. The absence of uncorrelated pairs could be induced by both, the global oscillatory trend that may be present in a majority of transcripts, or by common array-to-array noise. Quantile normalization and median polishing lead to bimodal correlation distributions with comparable numbers of correlating and anti-correlating pairs and many uncorrelated gene pairs (Figures 2I, 2J). This is explained by the overcompensation of the global oscillation with simultaneous introduction of anti-phase oscillation into the weakly or non-oscillatory expression profiles. This massive overcompensation is not observed for cLOESS (Figure 2L) which yields a unimodal symmetric distribution with a peak at zero. This indicates that a large amount of correlation in the dataset is being removed. This is again consistent with the decrease of the number of significant oscillators and with the dampening of the global diurnal trend. While LOS normalization is the only method which preserves the correlation and phase characteristics of the unnormalized data, it introduces a small number of anti- and non-correlating pairs. This is potentially due to the removal of the positive correlation cause by real array-to-array noise.

It has been noted before, that not only the background model, but also the type of data preprocessing can strongly affect the observed periodicity in a microarray dataset [35]. Normalization can significantly increase or decrease the number of oscillating transcripts. More importantly, however, normalization also introduces systematic biases into the transcripts peak phases, and can either reinforce or remove weak oscillatory signals that are in anti-phase to a global trend of the data. In the context of diurnal expression patterns, day-expressed transcripts may be converted to night-expressed ones and *vice versa*, depending on the choice for a normalization method. This fact can be expected to have extensive effects on subsequent analysis steps and the biological interpretation of results.

### Normalization and transformation shape clustering results

A common way of interpreting microarray expression data is clustering analysis. Clustering of data is often used to identify the temporal or functional organization of regulatory processes occurring, e.g., over one diurnal cycle [3,24]. As normalization methods can influence the expression profile similarity landscape on a global scale, we examined the impact of the normalization on the clustering analysis result. A large number of clusterings was generated, using all combinations of the described normalization methods, data transformations (12 m, std, DFT), and clustering algorithms. The obtained clusterings were analyzed for similarity.

This study focusses on a selection of seven popular clustering approaches based on diverse underlying principles which are described in more detail in the methods section. With K-means [36] and Partitioning Around Medoids (PAM) [37], the two well-established non-hierarchical clustering methods were included. The Self-Organising Tree Algorithm (SOTA) [38] and Hclust [39] represent the class of hierarchical methods. The Self-Organizing Maps (SOM) algorithm [40], an approach related to SOTA, was also included. Furthermore, two model-based methods Mclust [23] and flowClust [32] were considered. The flowClust clustering algorithm provides the Bayesian information criterion (BIC) as an estimate of the optimal number of clusters present in the data. As the BIC reached a plateau between eight to ten clusters for the different normalization-transformation combinations (Additional file 1: Figure S2), the following analysis is performed using clusterings with eight clusters.

The Euclidean distance and Spearman correlation coefficient were used separately as similarity measure if allowed by the clustering algorithm. Both measures differ fundamentally, since the Euclidean distance captures the absolute difference between each value of two time series whereas the Spearman correlation focusses on the relative differences.

**Figure 3:**
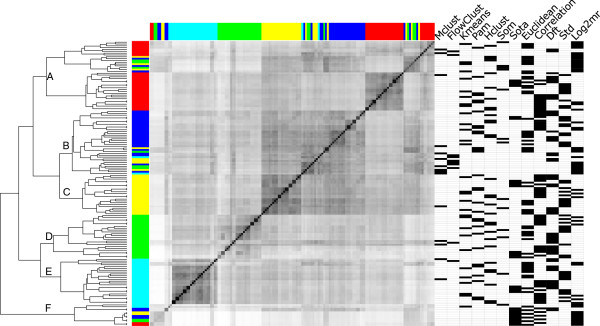
**Clustering results are determined by the normalization.** Pairwise similarity between all clusterings with eight clusters, similarity is measured using mutual information. White encodes minimal similarity over gray to black for maximal similarity. Rows and columns of the symmetrical matrix are ordered identically according to hierarchical clustering (Hclust, complete link method) of the similarities, represented as dendrogram on the left. The normalization method applied to the data before clustering is color-coded: no normalization - blue, median polishing - yellow, LOS - green, cLOESS - cyan, quantile normalization - red. The remaining processing steps (clustering algorithm, similarity measure, transformation) are represented as black bars in the corresponding column on the right. The column “correlation” marks the usage of the Spearman correlation coefficient as similarity measure except for clusterings obtained from SOTA, which only allows usage of the Pearson correlation.

To explore the large number of clusterings obtained from all combinations of the considered processing steps, the pairwise similarity between clusterings was measured using mutual information (MI, see Methods section for details). These pairwise similarities can be arranged in a matrix where each row and column corresponds to one individual clustering. When rows and columns are ordered identically this yields a diagonal matrix as shown in Figure 3. This similarity matrix can now be clustered again to reveal subgroups of particularly similar clusterings. We used a hierarchical clustering obtained with Hclust due to the intuitive dendrogram visualization.

We now asked whether the branches of the dendrogram correspond to particular parameters chosen to obtain the corresponding clustering. The specific parameter combination for each row of the similarity matrix is represented as annotation matrix on the right. This annotation matrix contains a column for every clustering algorithm, transformation, and similarity measure and black marks indicate usage in corresponding rows clustering. The normalization method is color-coded on the left/top of the similarity matrix.

Visual inspection of the normalization method pattern and the annotation matrix reveals six large subgroups A–F (Figure 3). Subgroup F constitutes the only clusterings that are dominated by the clustering algorithm. They are most distant to all other clusterings. Clusterings in this subgroup are derived using all normalization methods and mostly the SOTA and SOM algorithm. Large branch length and small numbers of leaves in the dendrogram show that clusterings in this subgroup are very diverse. Manual inspection reveals that all clusterings feature at least one small cluster (<10 genes). These observations indicate that these clusterings do not represent stable solutions and are disregarded. Inspection of the color-coded normalization methods (Figure 3, left) reveals that subgroups A to E are each dominated by one normalization method. That is, subgroup A contains mostly clusterings of quantile normalized data and subgroup B contains mostly clusterings of unnormalized data, but both contain a further sub-branch. Subgroups C, D, and E exclusively contain clusterings of median polished, LOS normalized, and cLOESS normalized data, respectively. Thus, the normalization method strongly influences the outcome of the clustering, overlaying potential differences in clustering algorithm or similarity measure.

Subgroups A and B, quantile-normalized and raw data, contain a sub-branch of clusterings that are based on other normalization methods. Inspection of the data transformation methods (Figure 3, right panel) reveals that these sub-branches contain mostly clusterings based on 12 m transformed data. We speculate, that the observed dominance of the 12 m transformation over the normalization method, i.e., higher clustering similarity due to transformation instead of normalization, reflects the design of the 12 m transformation to retain part of the amplitude information, whereas the std and our amplitude-scaling DFT transformation aim at its removal.

The similarity matrix in Figure 3 is shown only for clusterings with eight clusters, but the presented features are consistent within the range of five to fourteen clusters. Furthermore, the presented patterns are also found when using the normalized Variation of Information (Additional file 1: Figure S3) as clustering similarity measure. Application of the adjusted Rand index as clustering similarity measure also yields subgroups of clusterings of similarly normalized data (Additional file 1: Figure S4), but the hierarchical tree varies.

Comparison of the pairwise clustering similarity shows that the normalization method determines the clustering result more than any other step. Furthermore, the difference of the 12 m to the other transformations has a strong influence on the clustering. The 12 m removes the mean level but preserves amplitude differences in fluorescence intensity. Whether these differences are biologically meaningful or of technical character can not be determined due to the semi-quantitative nature of the microarray technology. It is therefore recommendable to focus on the pattern of change over time, which can be achieved by standardization or DFT with amplitude scaling [23]. In contrast, the choice of the clustering algorithm itself has the least impact on the obtained clustering result.

### LOS agrees best with biological knowledge

**Figure 4:**
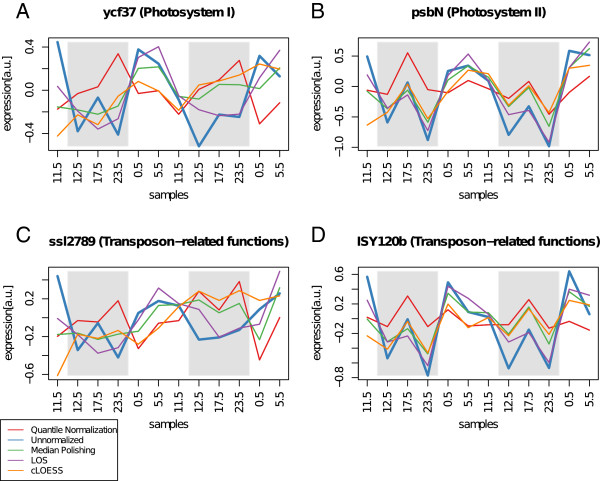
**Phase changes in high amplitude diurnal expression profiles due to normalization.** The expression profiles of four genes with clear diurnal oscillations before and after normalization with several methods using 12 m transformed data. The expression profiles are shown in different colors as provided in the legend. The gray shaded area marks the subjective night. The genes ycf37 (**A**) and psbN (**B**) are functionally associated with the photosynthesis and exhibit induced expression over the day. The expression phase *ϕ* after quantile normalization is shifted by ≈130°. The genes ssl2789 (**C**) and ISY120b (**D**) have transposon-related functions and are phase shifted by ≈160° after quantile normalization.

The implications of the observed normalization effects for the biological data interpretation are demonstrated for selected genes as well as the functional enrichment of a complete clustering result. First, we examined the set of significantly oscillating genes which exhibit large phase shifts after data normalization. As an example, the expression profiles of four such genes are shown in Figure 4. The LOS normalized profiles closely resemble the unnormalized profiles and exclusively dampens or remove expression spikes at the CT 17.5 samples. Whereas all genes exhibit an induction of expression over the day, application of quantile normalization always leads to a phase shift of ≈130−160° and, therefore, expression during the night as well as a dampening of the oscillation amplitude. Median polishing shows more diverse effects on the individual gene profiles. For gene ycf37 (ORF slr0171) shown in Figure 4A, median polishing preserves the oscillation phase in the first period, but severely attenuates oscillatory behavior in the second period. For gene psbN (ORF smr0009) and ISY120b (ORF sll1156) shown in panel B and D, the median polished profiles closely resemble the unnormalized profiles, while median polishing leads to no discernible oscillatory behavior for gene ssl2789 (C). Similar to median polishing, cLOESS shows diverse effects for the different genes.

For the gene ycf34, the first peak is preserved whereas the second period oscillation is removed. In case of the genes ISY120b and psbN, the cLOESS normalized profiles resemble the unnormalized and LOS normalized profiles, but feature oscillations with severely dampened amplitude. While on one hand, the diurnal oscillations of the raw data are entirely suppressed in the profile of gene ssl2789, on the other hand the amplitude of a negative anti-phasic spike at the first 0.5 CT sample is even increased.

As demonstrated, the choice of normalization methods can change the qualitative properties of the experimental data. While it is possible, that the global oscillatory trend is an experimental artifact and thus should be removed, this removal (e.g. by quantile normalization) leads to the conversion of day-active oscillators into night-active ones. Especially for the two photosynthesis-related genes ycf37 and psbN (Figure 4A, B) this is counter-intuitive and contradicts previous findings [41]. Only LOS normalization yields expression profiles which widely resemble the unnormalized profiles, while dampening the presumably noise-related peaks at both 17.5 CT samples. As already shown in the correlation distributions, cLOESS suppresses oscillatory behavior while preventing introduction of anti-phasic oscillations.

### Conservative normalization gives biologically reasonable results

**Figure 5:**
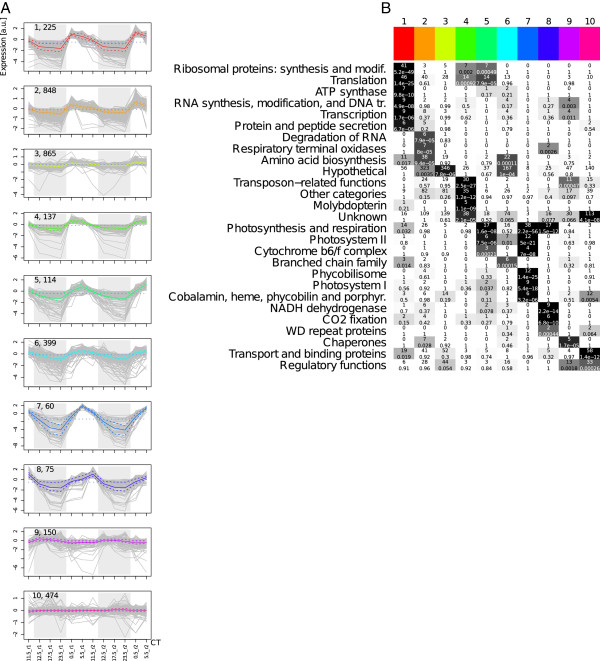
**Clustering after LOS normalization yields coarse biological program.** The clustering of LOS normalized DFT transformed data using the flowClust approach with ten clusters is shown in panel **A**. The gray lines represent individual gene profiles, the solid colored line marks the cluster mean profile, and the dashed colored lines mark the 5% and 95% quantiles. For visualization the 12 m transformed data are used. On the upper left corner of every profile plot, the cluster index is given followed by the number of genes in the corresponding cluster. The gray shaded area marks the dark period. The clusters are sorted by the mean phase angle *ϕ*. A graphical representation of the cluster-wise functional enrichment of the clustering shown in **A** is presented. The rows of this matrix correspond to biological functions whereas the columns correspond to clusters, where the color marks on the top match the colors used for the cluster mean profiles. The number of genes with the corresponding function is shown on the top of each cell and the enrichment p-value on the bottom. Furthermore, the enrichment p-value is color-coded in the cell background, marking highly significant enrichments in black and non-significant enrichments in white. The rows were rearranged to reveal the temporal ordering.

Finally, it remains to be shown that the presented data set and the processing provide a biologically reasonable picture. As demonstrated, the LOS normalization shows the least impact on the data and was consequently used in this analysis step. Visual inspection of clustering results revealed very good performance of flowClust with DFT transformation, where cluster-wise coherence of shape and phase of expression profiles were used as prominent criteria. From the range of optimal cluster numbers (8-10) according to the Bayesian information criterion as obtained from flowClust (see Additional file 1: Figure S2), we used ten clusters to ensure a finer resolution of the data for the following biological interpretation. Figure 5 A shows this clustering after reordering the clusters according to the mean expression phase *ϕ* of the corresponding cluster members. Functional category annotations for the enrichment analysis were obtained from the Cyanobase database [42]. For every individual cluster, the probability of the observed frequency of annotations was calculated assuming a hypergeometrical distribution (see Methods section for technical details). A visually enhanced version of the resulting table of enriched functional annotations for every cluster is shown in Figure 5B. To allow for comparison, the corresponding results for the other normalizations and unnormalized data are provided as Additional file 1: Figures S5–S8.

Most importantly, the three photosynthesis-related clusters 5,7, and 8 peak as expected in the morning, midday, and evening, respectively. The expression of components of the transcriptional and translational machinery in cluster 1 increases sharply during the DL transition. This could be explained by the extensive metabolic changes due the transition from respiration to photosynthesis as well as the induction of a variety of processes to utilize the readily available photosynthetic energy. Only with slight delay, the expression of amino acid biosynthesis related genes increases possible to provide the basic elements for protein synthesis. In contrast to protein synthesis, C*O*_2_ fixation related genes show an increased expression in the second half of the day (cluster 8). This behavior might reflect a separation between protein synthesis and cellular maintenance during the first half of the day and an accumulation of storage metabolites during the second half as preparation for the night as observed, e.g., in *Cyanothece sp.* ATCC 51142 [43]. The enrichment of genes with regulatory functions in the non-oscillating cluster ten is reasonable, since many regulatory mechanisms must be expected to respond to specific non-periodic cues.

## Conclusions

The expression of a large number of genes oscillates diurnally in a variety of cyanobacterial strains. In the microarray-based evaluation of diurnal patters in the transcriptome of the cyanobacterium *Synechocystis sp.* PCC 6803 presented here a large number of diurnally oscillating expression patterns was found in combination with a global diurnal oscillation. This global oscillation posed a problem for commonly used multi-chip normalization methods. Several methods that have been applied previously in a similar context attribute such a global oscillatory trend to technical variation and aim at its removal. We used several time series descriptors (phase, oscillatory p-value *p*_*osc*_) and clustering analyses to systematically compare the impact of four normalization methods on the presented dataset.

We found that the popular methods median polishing, quantile normalization and cyclic LOESS (cLOESS) normalization systematically change the expression phase of oscillating genes compared to the unnormalized data. This expression phase information is best preserved by the least oscillating set (LOS) normalization, which attributes changes in the least oscillating genes to technical variation and preserves the global oscillatory trend. Analysis of the expression profile correlation shows only minimal impact of the LOS normalization. In contrast, quantile normalization and median polishing strongly alter the original correlation structure by introducing anti-phasic oscillations. Only cLOESS suppresses oscillations without introducing anti-phasic ones. Moreover, the numbers of oscillating genes differ vastly between the different normalization methods. The reason for these normalization side effects is the oscillation in the mean transcript abundance. Only LOS normalization avoids the removal of this global trend and thereby avoids introduction of new anti-phasic oscillations or severe dampening of observed oscillators. On the other hand, LOS normalization may de-emphasize potential real but weak biological periodicities that are superimposed by the global trend, i.e., transcripts that may specifically peak during the night phase. The mechanism which leads to the oscillation in the mean transcript abundance, despite the consistent application of 1.5*μg* RNA on each individual microarray chip, may have several not mutually exclusive sources. Firstly, microarrays probe only a subset of the potentially expressed genomic sequences. A diurnal variation of the fraction of probed to non-probed transcripts in the total RNA extract may thus underlie our observation. Secondly, sequence properties such as the GC content introduce a bias into the resulting microarray signal. Strong overrepresentation of sequences with similar bias-introducing properties in the set of day- or night-expressed genes might therefore cause an oscillation. This explanation would predict the observation of a similar oscillation when using RNA-seq instead of microarrays, since sequencing-based techniques possess a similar bias. While the normalization of the microarray signal to the cell number via spike-ins proved useful to examine a global expression induction [18], this approach would not be sufficient in the presented case. Further experimental characterization of this diurnal trend is required to understand this phenomenon. It was shown that the result of a clustering analysis is governed by the choice of the normalization method rather than by the data transformation, similarity measure, or clustering algorithm. The only exception is the *log*_2_ mean ratio transformation, which emphasizes amplitude information more than the standardization and DFT transformation. Since this amplitude information can not be interpreted in a quantitative manner, it should be removed by standardization and DFT transformation to allow for exclusive clustering by the pattern of change. Comparison of existing biological knowledge shows that the combination of LOS normalization, clustering using flowClust and DFT transformation, and functional enrichment analysis of the resulting clusters outline the basic diurnal biological program of *Synechocystis sp.* PCC 6803. Other normalization methods cause large phase shifts or the attenuation of diurnal oscillations, which are in some cases inconsistent with biological knowledge.

In the light of these analyses, it is possible that the descriptions of large scale oscillatory gene expression and, in particular, expression timings in different cyanobacterial species are biased by the normalization methods employed in the analysis. To overcome this challenge, more robust multi-chip normalization methods must be considered when studying temporal expression organization. Importantly, the exact source of a diurnal trend in the total chip signal, despite experimental normalization, requires further experimental characterization.

## Methods

### The synechocystis sp. PCC 6803 time series expression dataset

*Synechocystis sp.* strain PCC 6803 was grown in BG11-medium [44] at 30°C under continuous illumination with white light of 120 *μ*mol of photons *m*^−2^*s*^−1^ and a continuous stream of air. The optical density of the culture was monitored by measuring the absorbance at 750 nm. Cultures were synchronized with three cycles of light/dark 12 h:12 h prior sampling. Aliquots were taken at OD750 0.5. Over a 24 h time course, 6 samples for RNA isolation were taken at the following time points: 30 minutes before and after light is switched off, (sample 1 - CT 11.5 and sample 2 - CT 12.5), 30 minutes before midnight (sample 3 - CT 17.5), 30 minutes before and after light onset (sample 4 - CT 23.5 and sample 5 - CT 0.5) and 30 minutes before noon (sample 6 - CT 5.5). Cells were filtered rapidly through Supor 0.45 m membrane filters (PALL), immediately stowed with TRIzol reagent (Invitrogen) and frozen in liquid nitrogen. Total RNA samples stored at -20°C were transferred directly to a 65°C waterbath for 5 minutes, mixed with 0.2 ml chloroform per ml of TRIzol and incubated for 15 minutes. The dissolving of the membrane and lyses of the cells were supported by vortexing. Centrifugation at maximum speed for 10 min at 4°C separated the phases. The RNA in the supernatant was precipitated by adding 0.5 ml of isopropanol per ml TRIzol used in the initial homogenisation. Two replicates were prepared from two synchronously growing cultures. The microarray design and hybridization procedure have been described previously [45]. The custom made Agilent single channel expression microarray holds probe sets for all annotated genes from the chromosome (NC_000911) as well as the seven plasmids. The detailed description of the employed microarray is deposited at gene expression omnibus (GEO) under the series identifiers GSE16162 and GSE14410. The extracted RNA was labeled directly for microarray hybridization to avoid labeling artifacts from reverse transcription and second strand synthesis during cDNA synthesis. The same amount of 1.5*μg* RNA was applied for every array, i.e. time point. The spot intensities were extracted with the ‘Agilent Feature Extraction Software 10.5.1.1’ using the Protocol GE1_105_Dec08. No background correction was performed. Probe summarization yields expression values for 8907 mRNAs, of which 3242 can be mapped onto protein coding genes located on the chromosome and 105 located on plasmid pSYSA. We selected only those genes for further analysis.

### Data transformation

The brightness of spots in a microarray experiment, from which the expression strength is derived, depends not only on the number of mRNAs in the sample, which is applied to the array chip. Large differences in hybridization energy and experimental effects like cross hybridization lead to expression values, which span several orders of magnitude and of which only relative changes for one probe set between the conditions can be interpreted. By the use of different transformations, it is common to bring raw expression data into the same order of magnitude. To allow for comparability, we also include the raw data in every step of our analysis.

#### Log2 mean ratio

The 12 m mean ratio is defined as

$$\begin{array}{ll} x^{\prime }=\text{lo}g_{2}\frac{x}{\bar{x}}\;, & \; \end{array}$$

where *x*, $\bar{x}$, and *x*^′^ denote the original time series, the average expression over the genes entire expression profile, and the transformed time series, respectively.

#### Standardization (Z transformation)

The standardization is defined as

$$\begin{array}{ll} x^{\prime }=\frac{\left(x-\bar{x}\right)}{\sigma_{x}}\;, & \; \end{array}$$

where *σ*_*x*_ denotes the standard deviation of the genes expression profile from its average, which is calculated as

$$\begin{array}{ll} \sigma_{x}=\sqrt{\frac{1}{N-1}\overset{N}{\underset{i=1}{\sum }}{\left(x_{i}-\bar{x}\right)}^{2}} & \; \end{array}$$

for an expression profile *x* of length *N*.

#### Discrete fourier transformation

A series of measurements *x*={*x*_0_,...,*x*_*N*−1_}, acquired at times {*t*_0_,...,*t*_*N*−1_}, can be approximated as a set of sine-functions with different frequency and amplitude. This transformation into frequency-space is done by applying the Discrete Fourier Transform (DFT) to each gene’s time series

$$\begin{array}{ll} X_{k}=\overset{N-1}{\underset{n=0}{\sum }}x_{n}e^{-2\mathrm{\pi i}\frac{\text{kn}}{N}}\;,\;k=\{0,\ldots ,N-1\}\;\;, & \; \end{array}$$

where *X* is a vector of complex numbers representing the decomposition. Each component *X*_*k*_ represents a sine with period *P*_*k*_=(*t*_*N*−1_−*t*_0_)/*k* where *X*_0_ represents the non-oscillating component or an offset from 0 of the time series. For each component *X*_*k*_ the amplitude *A*_*k*_ and the phase angle *ϕ*_*k*_ can be calculated as *A*_*k*_=|*X*_*k*_|/*N* and *ϕ*_*k*_=*tan*^−1^(*Im*(*X*_*k*_)/*Re*(*X*_*k*_)). Since the obtained spectrum is symmetrical relative to *k*=*N*/2, it can be restricted to 0<*k*<*N*/2 (in this case 0 to 6) without loss of any information. It must be noted that the computed phase angles *ϕ*_*k*_ provide a distorted measure of the diurnal expression time due to the non-equidistant sampling. However, the phase angles provide an excellent means to obtain a temporal order of oscillating expression patterns.

To be able to cluster these frequency spectra, we discard the uninformative non-oscillating component *X*_0_ and the highest frequency component *X*_6_ and create a series of values out of the 5 real and imaginary parts of the remaining frequency spectrum for every gene. This component omission can be interpreted as subtracting the mean for each gene’s time series. For the remaining components *X*_*k*_, the amplitude is scaled to emphasize the shape of the expression pattern instead of the absolute amplitude, which is less informative for microarray data. Therefore, the scaled amplitude *A*_*k*_ is the amplitude at component *k* divided by the mean of amplitudes at all other non-zero components, $a_{k}=A_{k}/{Ā}_{i\neq \{0,k\}}$.

### Detection of periodic expression profiles

As proposed previously, a permutation-based method is used to detect diurnal periodic expression profiles [20]. As diurnal periodicity is reflected in a large magnitude of the corresponding Fourier component *X*_*k*_, its significance can be assessed by the probability *p*_*osc*_ to observe *X*_*k*_ in a random permutation of the original time series. We, therefore, calculated the Fourier spectra of 100000 random permutations of each time series and calculated the empiric relative probability for each *X*_*k*_ to observe a Fourier coefficient equal or larger in a random permutation.

It must be emphasized that the Fourier transform uses a sine function as underlying model which in case of a sinusoidal expression profiles leads to a distinct peak in *X* at the corresponding frequency *k*. For periodic signals with non-sinusoidal shape, e.g. spike-shaped, the magnitude of the corresponding frequency component is distributed across the harmonic and neighboring frequency components. This hampers the detection of low-amplitude periodic non-sinusoidal profiles in comparison with sinusoidal profiles, since the lower magnitude of *X*_*k*_ receives a higher probability in the permutation background model.

### Data normalization

Strategies for the compensation of experimental variations in multi-chip experiments are generally considered necessary. Basis for such approaches are assumptions of similarity between different arrays in the same experiment.

The quantile-normalization approach by Bolstad *et al.*[46] assumes that the real distribution between the arrays is identical and only a small number of genes show differential expression due to the experiment. To perform the array-wide normalization we used the R-implementation in package limma [47] (normalizeBetweenArrays with method quantile).

Median polishing [48] is a classical method in exploratory data analysis. It is used within the RMA and GCRMA preprocessing protocols to summarize the probe sets. In this study, it is used to remove differences in the total median between individual arrays. We, thereby, illustrate the relaxation of the assumption of similar distribution shape, which is made in quantile normalization, while maintaining the assumption that the majority of genes are not differentially expressed.

With the LOESS normalization [34], another non-microarray specific normalization method finds wide acceptance. In this method, the observation of an expression amplitude-dependent non-linear relationship between multiple microarrays is accounted for using a polynomial correction function instead of a linear one for the equalization of two arrays. For the extension of this pairwise normalization, the gene-wise mean expression over all samples can be used as reference array for each individual sample array. In the work of Bolstad *et al.*[46], the cyclical application of the LOESS normalization was included, which we refer to in our comparison as cLOESS. We use the implementation in the R-package Limma using the method normalizeCyclicLoess using the default settings.

In addition, with the least oscillatory set (LOS) normalization we propose a method which is related to the least variant set normalization (LVS) [13] in its basic idea of selecting a subset of expression profiles for the fitting of a LOESS polynomial.

While LVS attempts to define a set of housekepping genes by finding profiles with minimal array-to-array variation (after partitioning the observed variation into array-to-array variation, within-probeset variation and residual variation), LOS follows a more intuitive approach. Here, housekeeping genes are defined as the set, which exhibits the least pronounced diurnal oscillations (measured by oscillatory p-value *p*_*osc*_). Defining the lower cutoff *p*_*osc*_>0.7 and considering all transcripts on the chip yields a LOS set of 1173 expression profiles. The global mean expression for each array is shown in Additional file 1: Figure S1 A together with the mean expression profiles of LOS sets of different size. The mean expression for each of these LOS profiles is used to fit a LOESS normalization curve to each individual array, which is then used to perform the normalization. For the presented dataset, LOS normalization leads to the dampening of the spike at the first CT 17.5.

### Clustering algorithms

From the plethora of clustering algorithms, which have been proposed for the clustering of expression data, we chose a diverse set of 7 methods which cover different principles of clustering.

#### K-means

The non-hierarchical K-means clustering algorithm is implemented in the R-function Kmeans (package: amap). In this function, 100 random starting sets of *k* cluster centers are used to run 1000 iterations of the Lloyd-Forgy algorithm [36] each. From the set of available distances measures, we chose the Euclidean distance and Spearman correlation coefficient *ρ*. In this case as in every following correlation coefficients have been transformed into a distance measure by:

$$\begin{array}{ll} \hat{\rho }=1-\rho & \; \end{array}$$

taking 1 minus the correlation coefficient.

#### Partitioning Around Medoids (PAM)

Similar to K-means, PAM is a non-hierarchical clustering algorithm that partitions the data by attempting to minimize the squared error of a distance measure [37]. In contrast to K-means PAM takes data points as cluster centers, which are then called exemplars or medoids. We are using the R-implementation pam (package: cluster) with Euclidean and Spearman correlation distance.

#### Hclust

The bottom-up hierarchical cluster [39] analysis included in this study is implemented in the R-function hclust (package: stats). The clustering is based on a set of dissimilarities between the samples. Here, we have used dissimilarities based on the Euclidean distance and the Spearman correlation coefficient together with Ward’s method [49].

#### Self-Organizing Maps (SOM)

The non-hierarchical Self Organizing Map (SOM) approach represents multidimensional data in a low-dimensional topological map. The grid used here is one-dimensional and the number of grid points equals the number of clusters [40]. The implementation of SOM in the R-function som (package: kohonen) [50] is used. During the training phase the data are presented for 3000 times to the network.The learning rate alpha is set to start from 0.5 and decreases linearly to 0.05 over the 3000 repetitions. As topology we chose a rectangular network with 1 by *k* nodes.

#### Self-organising tree algorithm (SOTA)

The top-down approach called self-organising tree algorithm or SOTA was proposed as strategy for phylogenetic reconstruction [38]. It has also been used to cluster microarray gene expression data [40]. In a top-down fashion, SOTA produces a hierarchical binary tree structure by repeatedly training a neural network and splitting the most diverse neuron into two neurons of the new network. We used the R-implementation clValid (package: clValid) with default parameters [38].

#### Mclust

We included a non-hierarchical model-based clustering approach using expectation maximization initialized by hierarchical clustering for parametrized Gaussian mixture models [23]. Each mixture component represents a cluster. The full set of 10 possible models is calculated for each number of clusters *k* and the model yielding the highest Bayesian information criterion (BIC) is selected. The R-implementation Mclust (package: Mclust) is employed with default parameters.

#### flowClust

As a second member of the family of model-based clustering methods we chose flowClust [32]. The main difference to Mclust is the usage of a multivariate t distribution as model for each cluster instead of a Gaussian distribution. We used the R-implementation flowClust (package: flowClust) with default parameters. The application of flowClust to standardized and unnormalized data prevented the convergence of the algorithm or lead to clusterings that include clusters of less than 10 genes. This suggests incompatibility of the algorithm to these transformations and justified the exclusion of these combinations from further analysis.

### Clustering comparison

#### Adjusted rand index

The Rand index [51] between two clusterings counts for all pairs in the dataset how often both are in the same cluster (*a*) or in different clusters (*b*) within both clusterings (agreement of clusterings). Also the number of disagreements in between all pairs is counted, i.e., for how many pairs both are in the same cluster in clustering 1, but not in clustering 2 (*c*) and vice versa (*d*). The counts are then combined to a score:

$$\begin{array}{ll} R=\frac{a+b}{a+b+c+d} & \; \end{array}$$

The adjusted Rand index furthermore accounts for similarities in the clusterings which are expected by chance. The adjusted Rand index values are of interval [0,1] where 1 is reached by maximally similar and 0 by maximally dissimilar clusterings. We use the R-implementation of the adjusted Rand index in function cluster.stats (package: fpc).

#### Mutual information

The mutual information is defined as

$$\begin{array}{ll} I(X,Y)=\underset{y\in Y}{\sum }\underset{x\in X}{\sum }p(x,y)\text{log}\left(\frac{p(x,y)}{p_{1}(x)p_{2}(y)}\right)\;, & \; \end{array}$$

where *p*(*x*,*y*) is the joint probability function for elements of the two clusterings *x*∈*X*,*y*∈*Y* and *p*_1_(*x*),*p*_2_(*y*) are the marginal probabilities for elements in the individual clusterings. The joint probability function is estimated by a contingency table whereas the marginal distributions are estimated by a histogram with each cluster being one bin. The mutual information values range from 0 for maximally dissimilar clustering to a maximum of the entropy of one clustering when both are identical. Therefore, the maximum mutual information increases with the cluster number enabling for a larger entropy value in a clustering. We used the R-implementation of the mutual information in function mi.empirical (package: entropy).

#### Normalized variation of information

The variation of information was proposed by Meila [52] is defined as follows:

$$\begin{array}{lll} \;\text{VI}(X,Y) & =H(X)+H(Y)-2I(X,Y)\; & \;\\ \text{nVI}(X,Y) & =\frac{\text{VI}(X,Y)}{H(X,Y)}\; & \; \end{array}$$

where *H*(*X*),*H*(*Y*) are the entropies of the individual clusterings, *I*(*X*,*Y*) is the already introduced mutual information. Instead of the variation of information *VI*(*X*,*Y*) we used the normalized variation of information to facilitate comparability between e.g. clusterings with different k. Values of the normalized Variation of Information are of interval [0,1] where 0 is reached by maximally similar and 1 by maximally dissimilar clusterings. We use the R-implementation of the VI in function cluster.stats (package: fpc) with subsequent normalization.

The construction of a clustering result comparison similar to Figure 3 is demonstrated in Additional file 2 using the statistical programming language R.

### Functional enrichment analysis

The functional enrichment analysis was performed using the gene annotations as provided by the Cyanobase database [42]. The overrepresentation of genes with a certain functional annotation was then computed with the R-library topGO [53], using the *classic* algorithm and the *Fisher* test statistic.

## Competing interests

The authors declare that they have no competing interests.

## Authors’ contributions

IA provided the biological samples. JG performed the microarray measurements. RL implemented the presented analysis, interpreted the data and wrote the manuscript. RM was involved in the developing of the ideas presented in this paper, the implementation of the analysis and the interpretation of the results. MB was involved in writing the manuscript. RS was involved in the developing of the ideas presented in this paper and the interpretation of the results. All authors read and approved the final manuscript.

## Supplementary Material

Additional file 1 **Supporting Information.** A document providing supplementary figures. Click here for file

Additional file 2 **R Script demonstrating application of the considered clustering algorithms.** A document, describing the application of clustering algorithms to time series expression data, using the statistical programming language R. Click here for file
