# Supplementary material for: How cyanobacteria pose new problems to old methods: challenges in microarray time series analysis
Source: BMC Bioinformatics. 2013 Apr 21;14:133. doi: 10.1186/1471-2105-14-133 (PMC3679775; doi:10.1186/1471-2105-14-133)
Supplement: Additional file 1 — Supporting Information. A document providing supplementary figures. [file 1471-2105-14-133-S1.pdf]

How cyanobacteria pose new problems to old methods: Challenges in microarray time series analysis

Robert Lehmann*[1] , Rainer Machné[3] , Jens Georg[4] , Manuela Benary[1] , Ilka Axmann[1,2] , Ralf Steuer[1]

[1]Institute for Theoretical Biology, Humboldt University Berlin, Invalidenstraße 43, D-10115, Berlin, Germany
[2]Institute for Theoretical Biology, Charité Universitätsmedizin, Invalidenstraße 43, D-10115, Berlin, Germany
[3]Institute for Theoretical Chemistry, University of Vienna, Währingerstraße 17, A-1090, Vienna, Austria
[4]Faculty of Biology and Freiburg Initiative in Systems Biology, University of Freiburg, Schänzlestraße 1, D-79104, Freiburg, Germany

Email: Robert Lehmann*- r.lehmann@biologie.hu-berlin.de; Rainer Machne - raim@tbi.univie.ac.at; Jens Georg - jens.georg@biologie.uni-freiburg.de; Manuela Benary - manuela.benary@cms.hu-berlin.de; Ilka M. Axmann - ilka.axmann@charite.de; Ralf Steuer - ralf.steuer@hu-berlin.de;

*Corresponding author

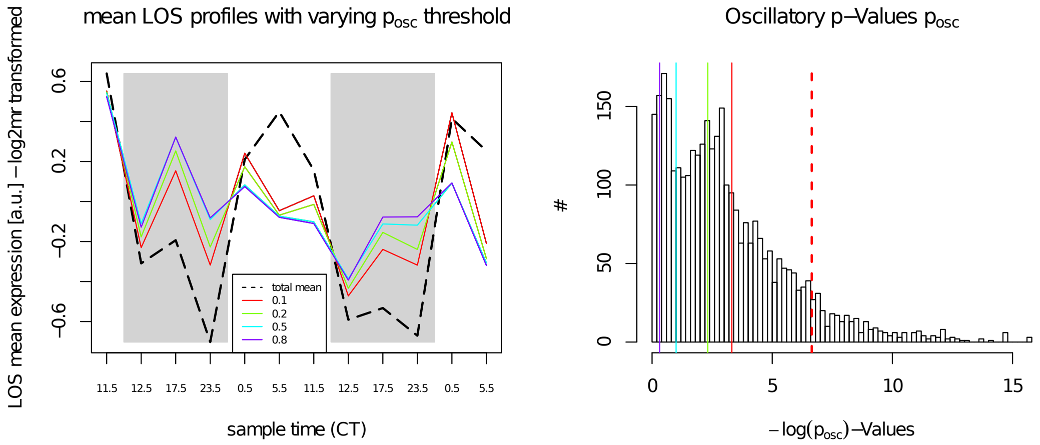

Figure 1: Global expression oscillation in unnormalized data (A, black dashed line). Mean profiles of gene sets used for LOS normalization using different cutoffs for p_{osc}. (B) The distribution of p_{osc} in the complete dataset with cutoffs indicated with identical colors to (A). The red dashed line marks the significance level of 0.001.

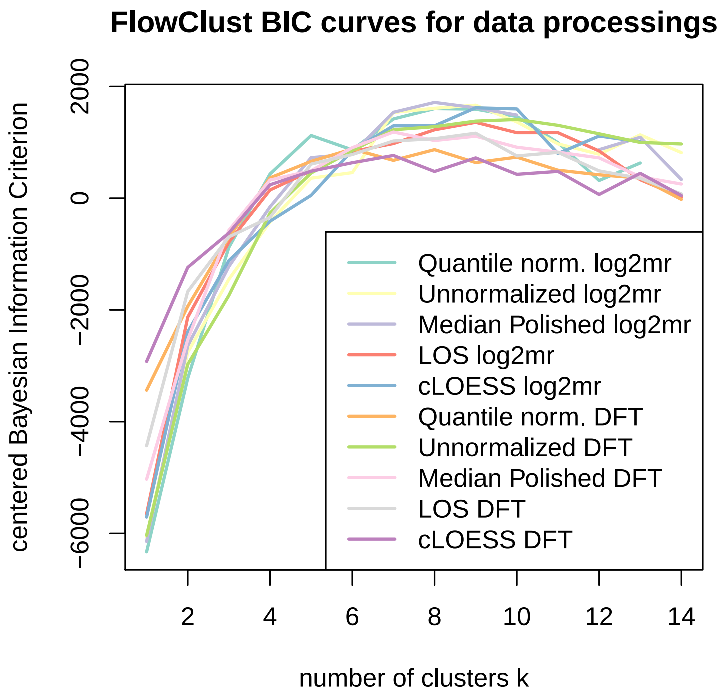

Figure 2: Number of Clusters in the Dataset. Bayesian information criterion for clusterings of data with different normalization and transformation as provided by FlowClust.

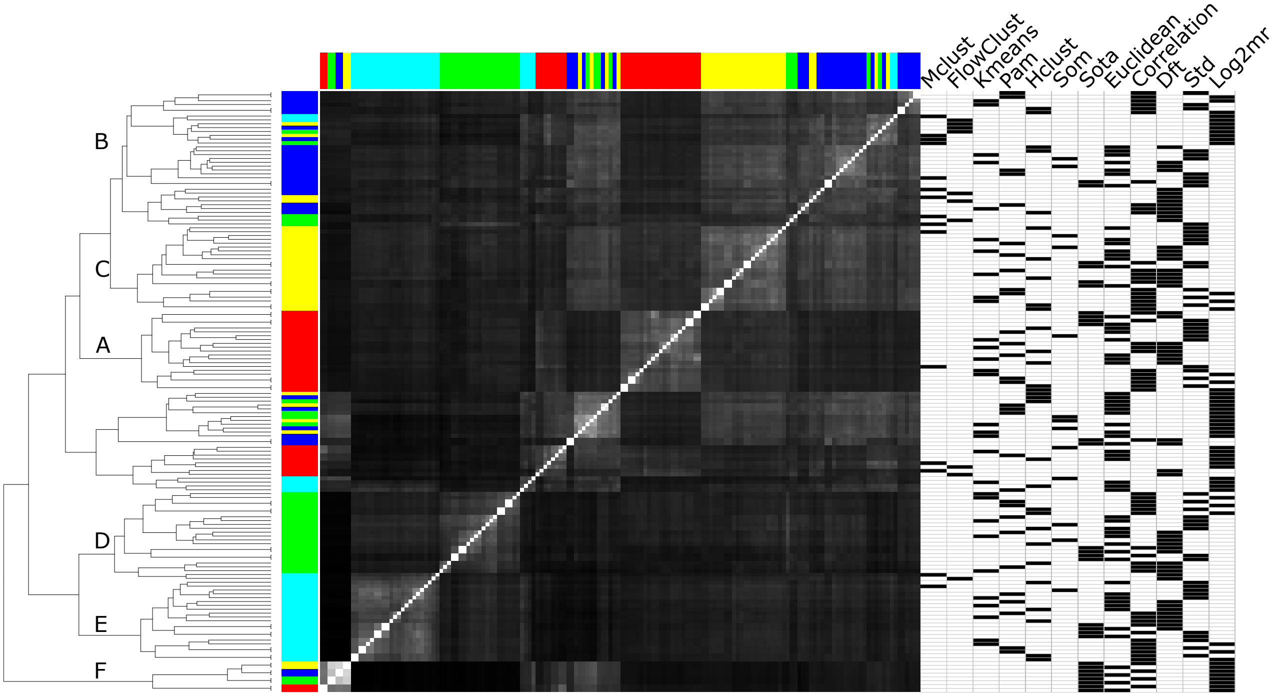

Figure 3: Comparing Clustering Results with the normalized Variation of Information between all clustering combinations with k=8. The visualization is identical to Figure 3.

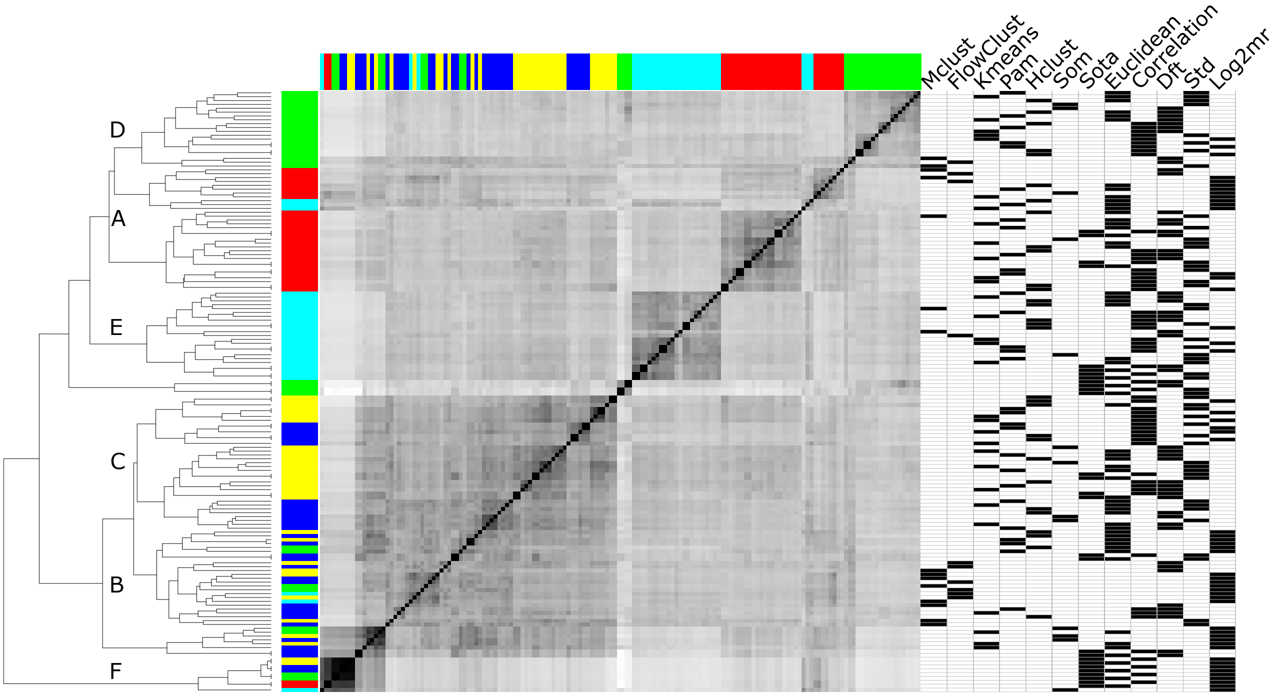

Figure 4: Comparing Clustering Results with the adjusted Rand index between all clustering combinations with k=8. The visualization is identical to Figure 3. The dendrogram of clusterings varies in comparison to the similarity matrices using mutual information (Fig.3) and normalized Variation of Information (Fig.S3). Subgroup A contains only a small subbranch of three cLOESS normalized clusterings. The remaining clusterings of this subbranch are moved into subgroup B, which is consequently more diverse.

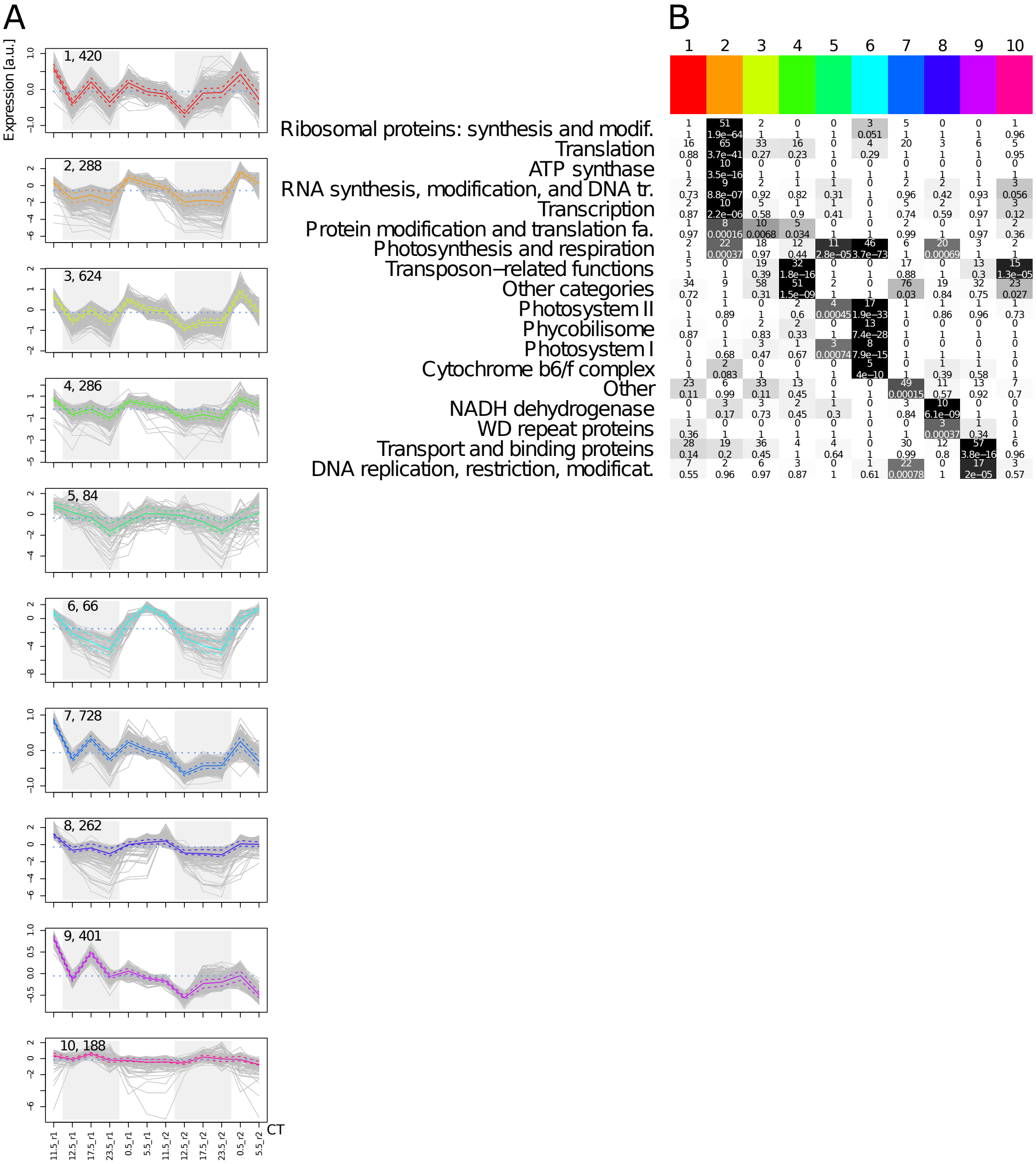

Figure 5: A) Unnormalized data clustered using flowClust with DFT transformation and ten clusters. The clusters are sorted by phase and profiles plotted using log_2 mean ratio transformed data.In each cluster plot, the cluster index followed by the number of genes in the corresponding cluster are shown in the top left corner. Subjective night is marked by a gray background. The sample times are provided in circadian time (CT) for cluster ten and are identical for all clusters. B) Functional enrichment result for each cluster in panel A. On top of the matrix the cluster indices are shown together with a color mark that corresponds to the color in A. On the left of the matrix the enriched functional categories are shown, sorted by cluster. Only significantly enriched categories are shown ($p < .05$). The p-value is shown on the bottom of every cell, the number of genes which are in the corresponding cluster and associated with the corresponding function is shown on top of each cell. Additionally, the enrichment p-value is color-coded in the background of each cell with black marking highest significance and white as lowest.

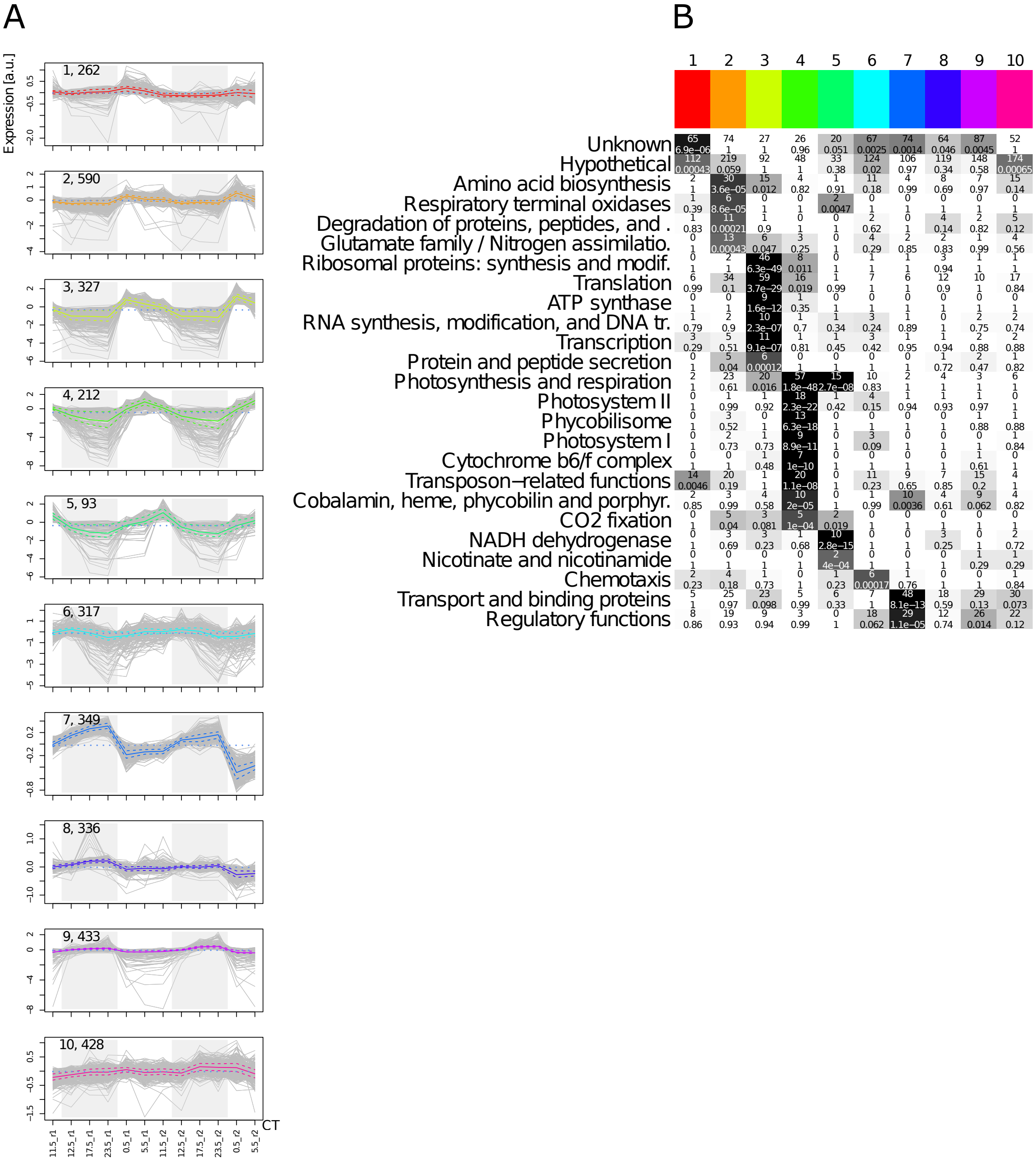

Figure 6: Clustering and functional enrichment for median polished data. The visualization is identical to Figure S5.

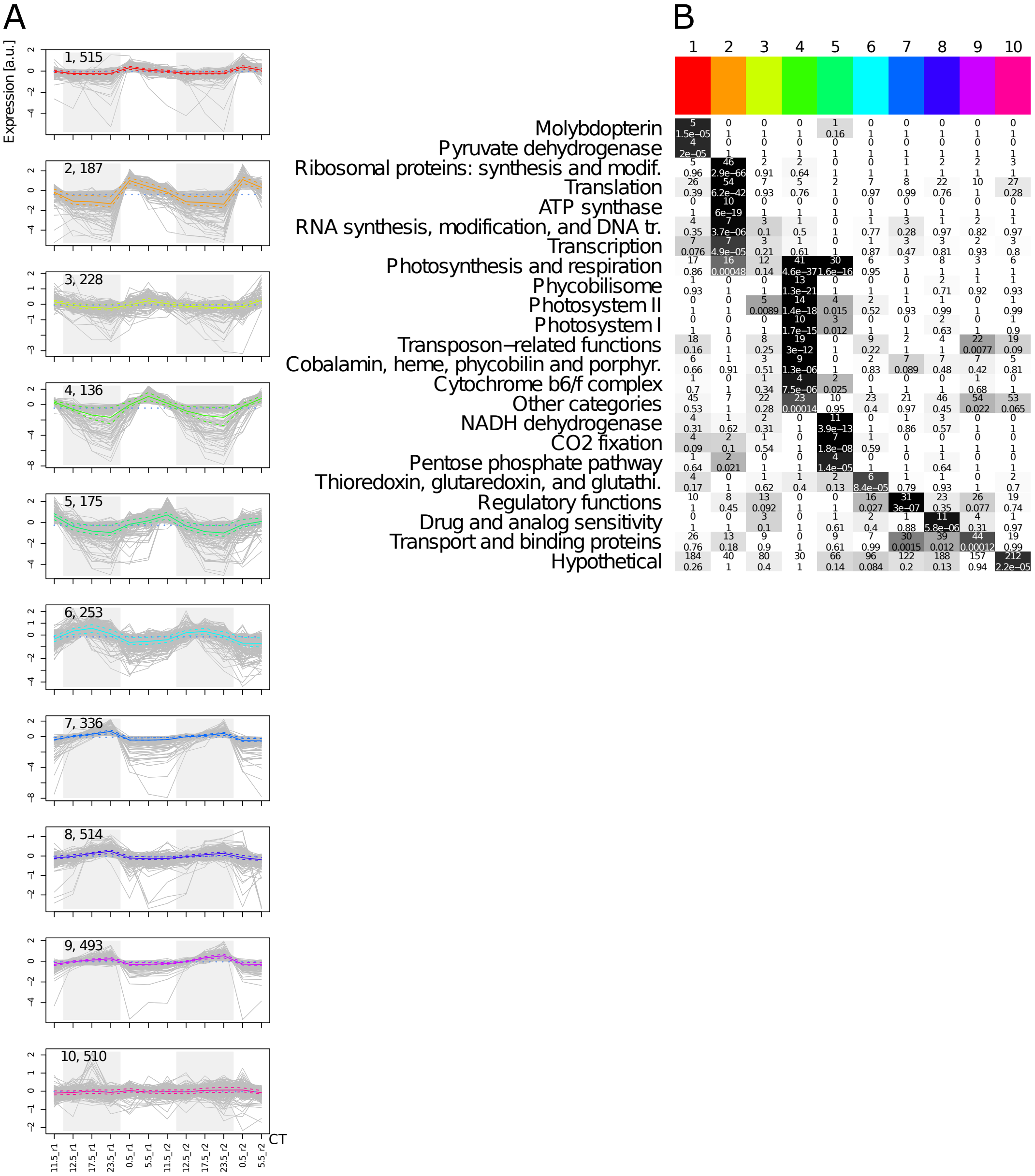

Figure 7: Clustering and functional enrichment for quantile normalized data. The visualization is identical to Figure S5.

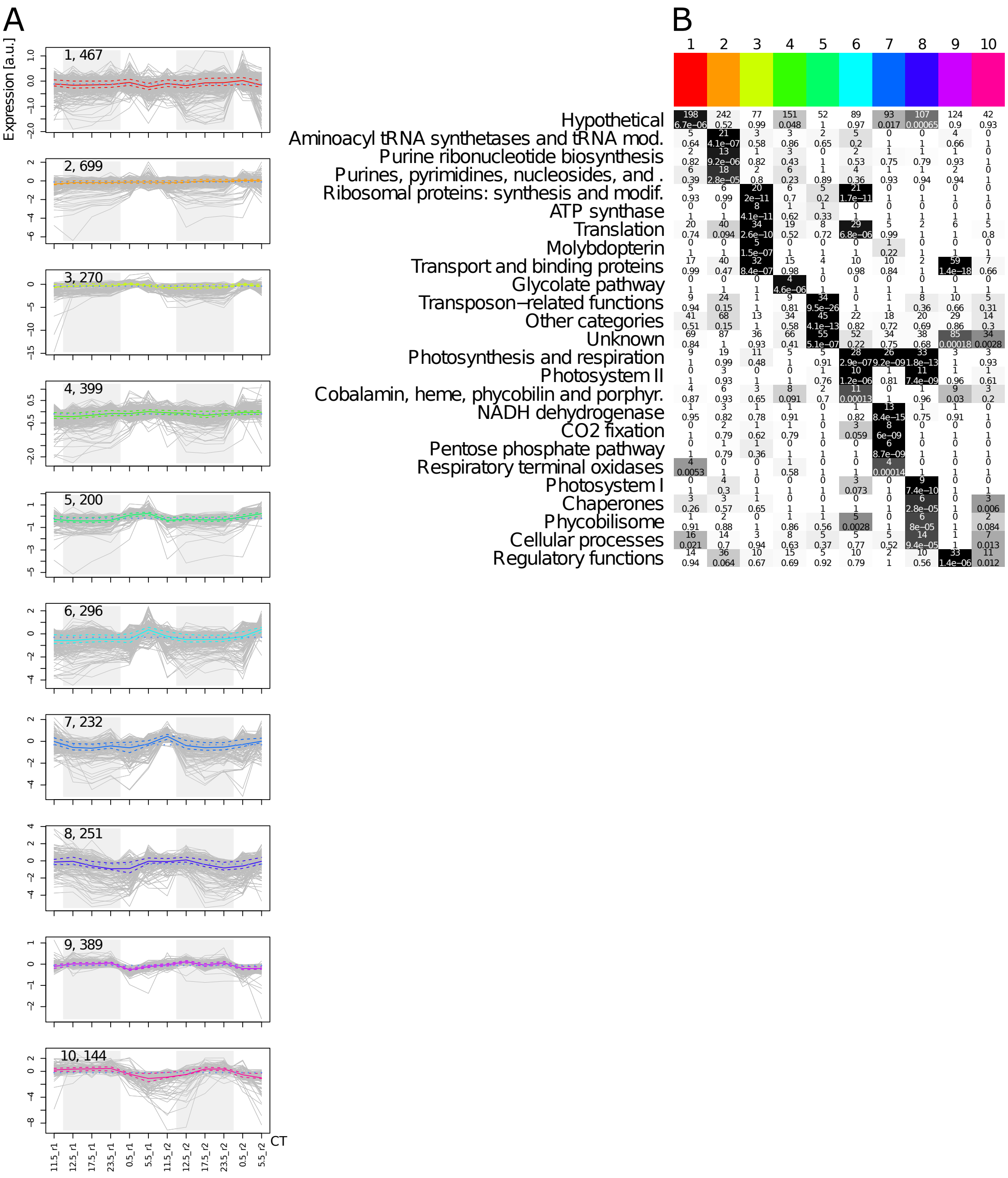

Figure 8: Clustering and functional enrichment for cyclic LOESS normalized data. The visualization is identical to Figure S5.
